# Role of Oxidative Stress in Thyroid Hormone-Induced Cardiomyocyte Hypertrophy and Associated Cardiac Dysfunction: An Undisclosed Story

**DOI:** 10.1155/2015/854265

**Published:** 2015-06-04

**Authors:** Mohammad T. Elnakish, Amany A. E. Ahmed, Peter J. Mohler, Paul M. L. Janssen

**Affiliations:** ^1^Department of Physiology and Cell Biology, College of Medicine, The Ohio State University, Columbus, OH 43210, USA; ^2^Dorothy M. Davis Heart & Lung Research Institute, The Ohio State University, Columbus, OH 43210, USA; ^3^Department of Pharmacology and Toxicology, Faculty of Pharmacy, Helwan University, Cairo, Egypt

## Abstract

Cardiac hypertrophy is the most documented cardiomyopathy following hyperthyroidism in experimental animals. Thyroid hormone-induced cardiac hypertrophy is described as a relative ventricular hypertrophy that encompasses the whole heart and is linked with contractile abnormalities in both right and left ventricles. The increase in oxidative stress that takes place in experimental hyperthyroidism proposes that reactive oxygen species are key players in the cardiomyopathy frequently reported in this endocrine disorder. The goal of this review is to shed light on the effects of thyroid hormones on the development of oxidative stress in the heart along with the subsequent cellular and molecular changes. In particular, we will review the role of thyroid hormone-induced oxidative stress in the development of cardiomyocyte hypertrophy and associated cardiac dysfunction, as well as the potential effectiveness of antioxidant treatments in attenuating these hyperthyroidism-induced abnormalities in experimental animal models.

## 1. Introduction

Oxidative stress is an expression describing a state of elevated reactive oxygen species (ROS) levels. ROS are reactive chemical entities including (1) free radicals such as superoxide (O_2_^∙−^), hydroxyl (^∙^OH), and nitric oxide (NO^∙^) and (2) nonradical derivatives of O_2_, such as hydrogen peroxide (H_2_O_2_) and peroxynitrite (ONOO^−^). In general, ROS control and/or are involved in several physiological processes, including host defense, biosynthesis of hormones, fertilization, and cellular signaling. However, ROS also have a high reactivity potential and thus may lead to oxidative damage to proteins, lipids, and DNA, resulting in cellular dysfunction [1]. The cellular protective mechanism against ROS damage comprises a number of enzymatic and nonenzymatic antioxidants that are capable of scavenging free radicals and preventing them from causing deleterious effects under physiological conditions [2]. Examples of enzymatic antioxidants are glutathione reductase (GR), glutathione peroxidase (GPx), glutathione-S-transferase (GST), catalase (CAT), and superoxide dismutase (SOD), whereas examples of nonenzymatic antioxidants include vitamins E and C, *β*-carotene, ubiquinone, lipoic acid, urate, and glutathione (GSH). Additionally, GSH is a reducing substrate for GPx enzymatic activities, and thioredoxin (Trx) and Trx reductase catalyze the restoration of numerous antioxidant molecules [3, 4]. When this cellular balance between ROS generation and antioxidant capacity is disrupted, oxidative stress develops [5]. This phenomenon has been linked to various pathological conditions [6, 7] including hyperthyroidism, the increased production of thyroid hormones (THs) [8].

The general actions of the THs (triiodothyronine (T3) and thyroxin (T4)) can be classified into two main categories: (1) growth and development regulation and (2) metabolism regulation which is directly coupled to ROS generation. The overall balance arising from the stimulation of both generation and abolition of ROS by THs entails a net increase in oxidative stress, as estimated by products of cellular damage such as lipid peroxidation. The extent of oxidative stress evoked by THs differs widely among tissues, with the greatest effects on the cell types that are more metabolically responsive to THs such as liver, red oxidative muscle fibers, lymphoid tissue, and heart [9].

The goal of this review is to shed light on the effects of THs on the development of oxidative stress in the heart along with subsequent cellular and molecular changes. In particular, we will review the role of THs-induced oxidative stress in the development of cardiomyocyte hypertrophy and associated cardiac dysfunction, as well as the potential effectiveness of antioxidant treatments in attenuating these abnormalities following experimental hyperthyroidism.

## 2. Thyroid Hormones and the Heart

**Figure 1:**
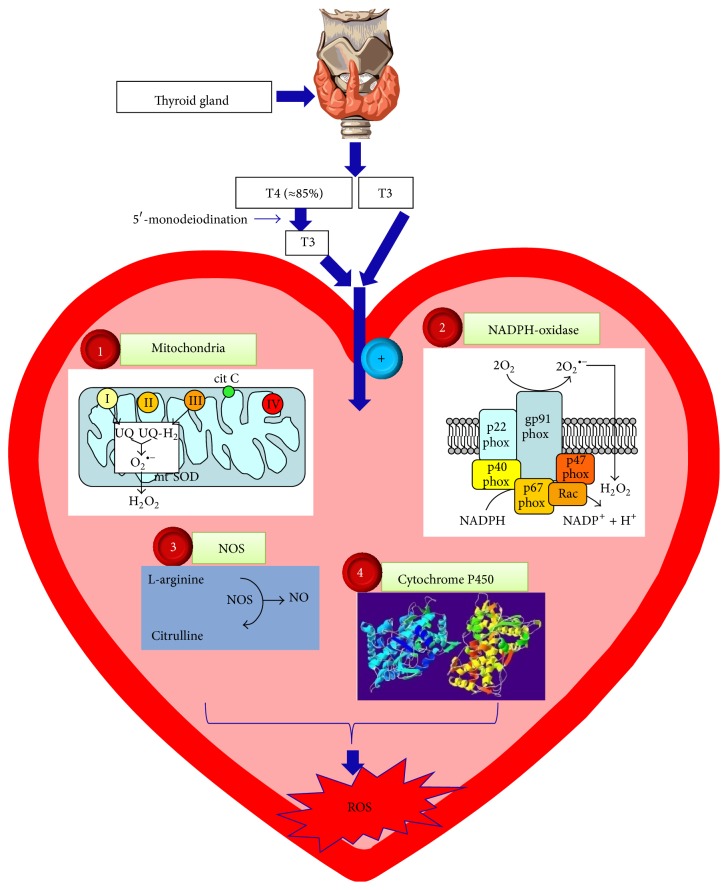
Potential sources of reactive oxygen species (ROS) in hyperthyroid hearts: T4: thyroxin; T3: triiodothyronine; (1) mitochondria; (2) NADPH- (nicotinamide adenine dinucleotide phosphate-) oxidase; (3) NOS: nitric oxide synthase; (4) cytochrome-P450; +: activation. Representative image of thyroid gland is copied from Wikipedia under the Creative Commons Attribution-Share Alike 3.0 Unported license, which allows sharing and/or remixing. Representative images of mitochondria, NADPH-oxidase, and NOS were adapted from Novo and Parola [65]: “Redox Mechanisms in Hepatic Chronic Wound Healing and Fibrogenesis,” licensee BioMed Central Ltd. This is an open access article distributed under the terms of the Creative  Commons  Attribution  License, which permits unrestricted use, distribution, and reproduction in any medium, provided the original work is properly cited. Representative image of cytochrome-P450 is copied from Wikipedia under the terms of the GNU Free Documentation License, Version 1.2, that allows copying, distribution, and/or modification.

**Figure 2:**
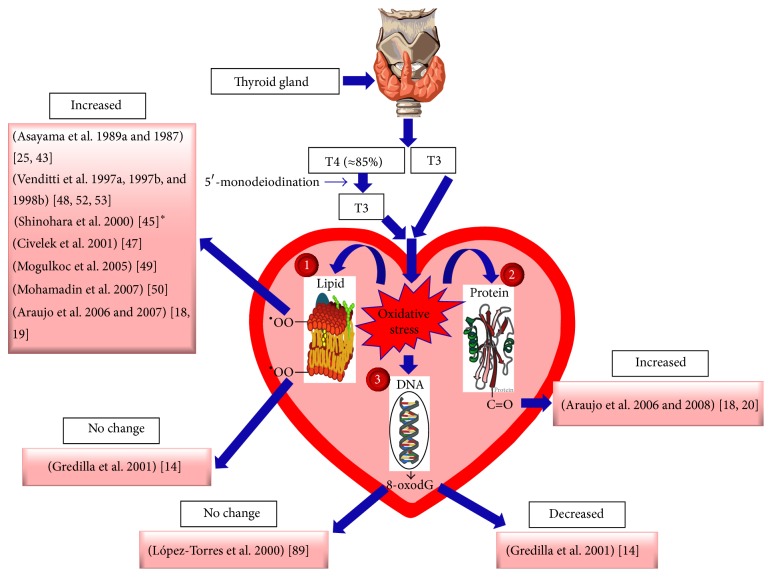
Markers of oxidative damage in the hyperthyroid hearts. Oxidative damage of (1) lipid as assessed by measuring by-products of lipid peroxidation such as thiobarbituric acid reactive substances (TBARS), hydroperoxides, chemiluminescence, and/or N^Σ^-(malondialdehyde)lysine (MDA), (2) protein as assessed by estimating protein-bound carbonyls (C=O), and (3) genomic DNA estimated as 8-oxo-7,8-dihydro-2′-deoxyguanosine (8-oxodG).  ^*∗*^In this study [45], old rats showed increased lipid peroxidation; however, young rats displayed no change. Representative images of thyroid gland, DNA, lipid, and protein are copied from Wikipedia under the Creative Commons Attribution-Share Alike 3.0 Unported License, which allows sharing and/or remixing.

**Figure 3:**
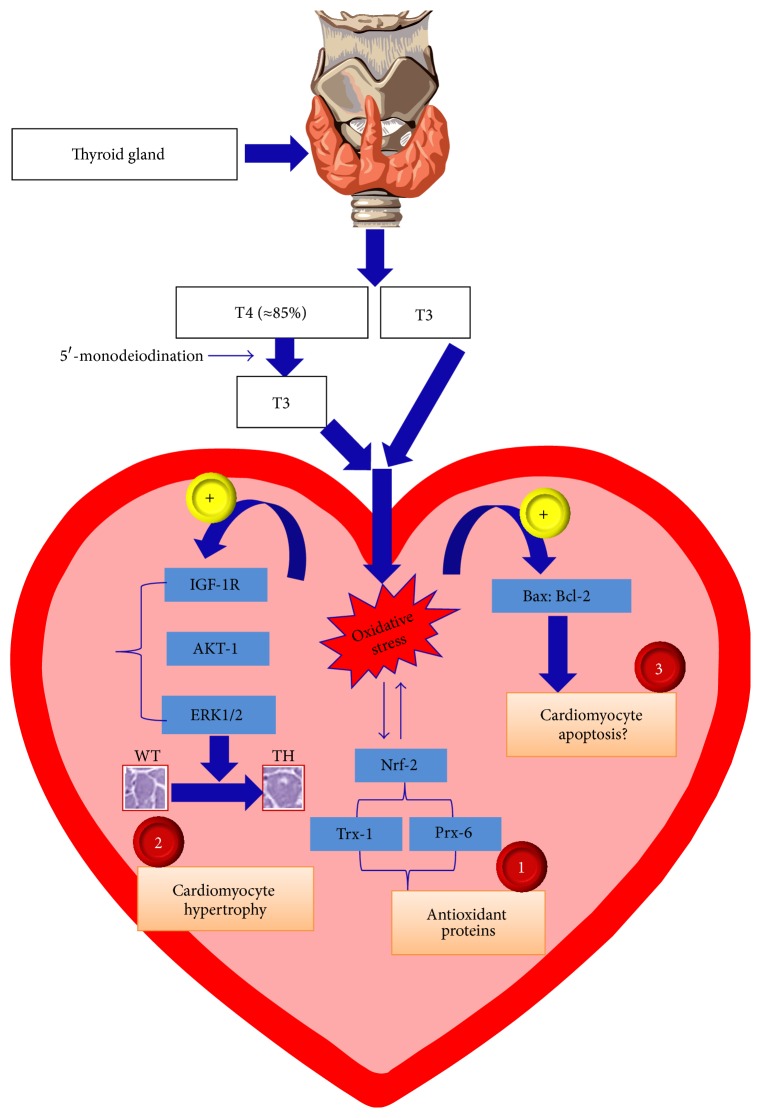
Molecular changes in the hyperthyroid hearts in response to increased oxidative stress. This includes main (1) antioxidant, (2) hypertrophic, and (3) apoptotic signaling activated by oxidative stress in. T4: thyroxin; T3: triiodothyronine; Nrf-2: NF-E2-related factor 2; Trx: thioredoxin; Prx: peroxiredoxin; IGF-IR: insulin growth factor-I receptors; AKT-1 (PKB): protein kinase B; ERK: extracellular regulated kinase; WT: wild-type; THs: thyroid hormones; Bax: Bcl-2: Bcl-2 family proteins where Bax is proapoptotic while Bcl-2 is antiapoptotic; +: activation; ?: not shown in this study. Representative image of thyroid gland is copied from Wikipedia under the Creative Commons Attribution-Share Alike 3.0 Unported License, which allows sharing and/or remixing. Images of cardiomyocytes from wild-type (WT) and thyroid hormone- (TH-) treated mouse hearts are adapted from Elnakish et al. 2012 [16].

**Figure 4:**
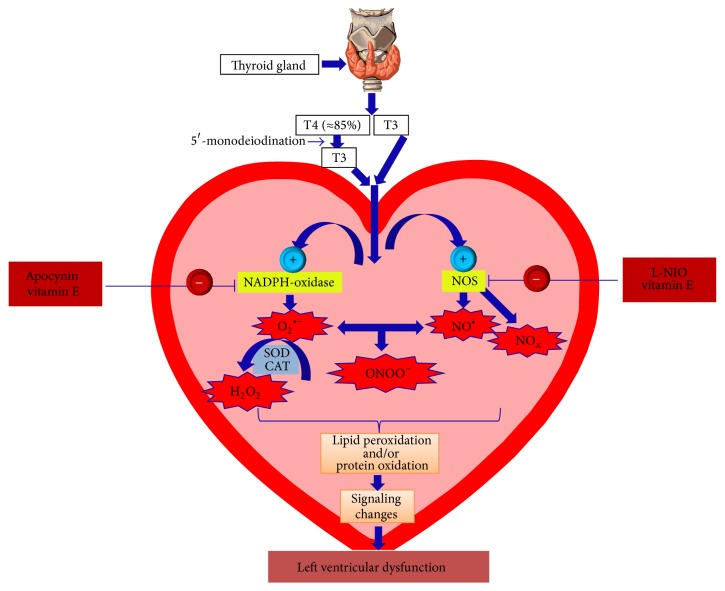
Putative mechanism of oxidative stress-induced left ventricular dysfunction in the hyperthyroid hearts. T4: thyroxin; T3: triiodothyronine; NADPH: nicotinamide adenine dinucleotide phosphate; NOS: nitric oxide synthase; O_2_^∙−^: superoxide; NO^.^: nitric oxide radical; H_2_O_2_: hydrogen peroxide; ONOO^−^: peroxynitrite; SOD: superoxide dismutase; CAT: catalase; L-NIO: N^5^-(1-iminoethyl)-L-ornithine dihydrochloride; +: activation; −: blocking. Representative image of thyroid gland is copied from Wikipedia under the Creative Commons Attribution-Share Alike 3.0 Unported License, which allows sharing and/or remixing.

While THs impact nearly all organ systems, the heart acts in response to minimal alterations in the serum levels of THs [10]. The thyroid gland principally secretes T4, which is converted to T3 by 5′-monodeiodination in liver, kidney, and skeletal muscle. The heart depends largely on serum T3 due to the lack of significant intracellular deiodinase activity in the cardiomyocytes, and it seems that T3, but not T4, is moved into the cardiomyocytes [11] (Figures 1–4). In the heart, THs are consistently known to induce cardiomyocyte hypertrophy [12–29]. Hypertrophy can be a compensatory response to enhance contractility and preserve cardiac output, exclusive of undesirable pathology. Nevertheless, persistent stress can drive this compensatory process into a decompensated state, with reflective alterations in gene expression profile, contractile dysfunction, and extracellular remodeling [1].

Generally, improved cardiac function is the most documented upshot of hyperthyroidism [12]. Nevertheless, it has been reported that TH-induced cardiac hypertrophy is linked to an initial increase in cardiac function followed by a reduction in cardiac performance signifying the harmful effects of chronic hyperthyroidism [13]. We have shown that a T4 dose of 200 *μ*g/kg/day for two weeks resulted in physiologic cardiac hypertrophy and preserved cardiac function in mice [15, 16], in contrast to pathologic cardiac hypertrophy with decreased cardiac function at higher T4 dose (500 *μ*g/kg/day) [17]. A similar myocardial dysfunction has been reported in the hearts of hyperthyroid rats [18–20]. Furthermore, dilated cardiomyopathy in which hyperthyroidism was the primary cause has been reported in animals after prolonged T4 treatment [21], as well as in human patients [30–33] indicating that excess THs can be a risk factor for human heart failure.

Primarily THs act through binding to nuclear receptors that promote or repress gene transcription. There are numerous cardiac genes identified as targets for transcriptional activation by THs, such as *α*-myosin heavy chain (*α*-MHC), sarcoplasmic reticulum calcium-activated ATPase (SERCA2), Na-K-ATPase, *β*-adrenergic receptor, cardiac troponin I, and atrial natriuretic peptide [34–39]. On the contrary, other genes are identified as targets for transcriptional repression, such as *β*-myosin heavy chain (*β*-MHC) [40]. A growing body of evidence suggests that a changed thyroid status in patients with cardiovascular diseases could amend gene expression in the heart and result in decreased cardiac function [41]. THs have also been proposed to act through a nongenomic mechanism, which can occur rather rapidly through binding to a membrane receptor to activate signaling. Thus, cardiac hypertrophy/dysfunction could also be the result of activating signaling pathways through such nongenomic mechanisms where oxidative stress and ROS may serve as potential modulators of this response in hyperthyroidism [22, 24, 42].

## 3. Sources of Increased Oxidative Stress in the Hyperthyroid Heart

The heart constantly produces O_2_ radical derivatives owing to its high bulk of active mitochondria which provide ATP, mainly to maintain cardiac contractile function. Furthermore, the heart, which is similar to muscle tissues in general, has predominantly low levels of antioxidants, and its postmitotic nature makes the repair of tissue damage more difficult [14]. Thus, the prosperity of data indicates that many harmful cellular phenotypes detected in hypertrophied and failing myocardium are accredited to oxidative stress, as we reviewed before [1].

THs are the most significant regulator of the basal metabolic state and oxidative metabolism [8]. Although controversy exists as to whether hyperthyroidism is coupled to an increase or a decrease in the antioxidant enzyme activities, experimental studies and epidemiological data propose that hyperthyroidism is linked to a common rise in tissue oxidative stress [2]. In this context, increased oxidative stress in the hyperthyroid heart has been consistently reported [43–53]. However, there are remaining discrepancies in the changes of the antioxidant activities observed in these hearts (Table 1). These discrepancies have been attributed to differences in animal age, treatment period, iodothyronine used (T3 or T4), or combination of some of these parameters [54]. For instance, total SOD was found to increase in the hearts of young but not old hyperthyroid rats. Conversely, cardiac GPx activity was found to decrease in the hearts of old but not young hyperthyroid rats [45]. On the other hand, Fernandes et al. found no significant differences in the cardiac Trx or GSH activities after 2-week treatment of T4 [24]; yet, the same group reported increased Trx [22] but decreased GSH [18–20, 22] activities in the hyperthyroid hearts after 4-week treatment in the same model. Additionally, it was reported that T4 [26] but not T3 [48, 53] decreases the cardiac GR activity. This could also be due to the differences in the treatment periods where comparable doses of both T3 [48, 53] and T4 [26] were injected for 10 days and 6 weeks, respectively. However, this is still inconsistent with the same iodothyronine treatment, and a higher dose of T4 for the relatively long period of 4 weeks was shown to increase such GR activity in the heart [22]. Furthermore, activities of different antioxidants were shown to vary in the same models under the same treatment conditions as shown in Table 1. Largely, these controversies may support the hypothesis that antioxidant levels may not primarily be related to oxidative metabolism in hyperthyroidism [52].

ROS can be produced in the heart by various potential sources such as mitochondria, NADPH-oxidase, uncoupling of NO synthase (NOS), xanthine oxidase, cytochrome-P450, and autoxidation of catecholamines [41]. In regard to mitochondria, increased mitochondrial-generated ROS has been demonstrated in cardiomyocytes from experimental models of heart failure or myocardial infarction [57, 58]. Notably, one of the key effects of THs is to enhance mitochondrial respiration through changing the number, as well as the activity, of several complexes in the mitochondrial respiratory chain [59]. Hastened mitochondrial electron transport achieved by TH-induced hypermetabolic state leads to the enhanced O_2_^∙−^ production, which in turn can lead to the generation of many other ROS [60, 61]. THs also regulate the synthesis of nuclear- as well as mitochondrial-encoded mitochondrial proteins via a nuclear mechanism [62]. Regardless of a decline in the number of mitochondria per cell in the hyperthyroid heart [63], there is a rise in respiratory chain proteins of the mitochondria [64]. These proteins can significantly contribute to the TH-provoked stimulation of mitochondrial respiration [59, 64] and cause enhanced ROS generation [53]. Effectively, Asayama et al. reported increased mitochondrial oxidative metabolism in hypertrophied hyperthyroid rat hearts and proposed a key role for this observation in TH-induced myocardial dysfunction [25, 43, 44].

Similarly, NADPH-oxidase, through redox-sensitive signal transduction, has been presented as a key player in the pathogenesis of several aspects of cardiac remodeling and its antecedent conditions both in human patients and in animal heart failure models [1]. Recently, the involvement of NADPH-oxidase-mediated ROS generation in the TH-induced oxidative stress and associated cardiac hypertrophy/dysfunction has been reported [23, 66].

NO, which is generally considered as an essential signaling molecule in normal cardiac physiology having a protective role in cardiac diseases, can also exert cytotoxic effects under settings of increased oxidative stress [67]. Under these settings, NO can interact with O_2_^∙−^ to generate ONOO^−^, destroying cellular functions and disabling the antioxidants such as SOD, CAT, and GPx, by interacting with their hydrosulfide groups. In addition, excessive NO can swiftly be oxidized into nitrogen dioxide, which operates as a catalyst in the polyunsaturated fatty acids lipid peroxidation process, consequently peroxidizing cellular membranes [41]. During increased oxidative stress, generation of further ROS could also be achieved by uncoupled NOS as a result of the BH4 oxidation, an essential cofactor of NOS [68]. In this regard, eNOS uncoupling was proposed to play a role in the LV remodeling secondary to chronic pressure overload in mice [69]. Furthermore, increased expression and activity of iNOS and nNOS along with NO overproduction have been reported in the failing myocardium as well as in different heart failure models [67]. A correlation between THs and cardiac NOS/NO has been frequently reported. Indirect evidence has revealed that generation of NO^.^ rises in hyperthyroid heart [70, 71]. Quesada et al. also reported increased NOS activity in the left ventricle (LV) of the hyperthyroid rats [72]. In the absence of autonomic influences, THs were shown to modulate the intrinsic heart rate through a mechanism that entails, at least in part, the NO pathway [73]. Interestingly, Araujo et al. have reported direct evidence of the key role of the NO pathway in TH-induced cardiac hypertrophy and cardiac dysfunction. In their studies, they showed increased NO metabolites (NO_*x*_) as well as increased activities of all NOS isoforms in the hearts of the hyperthyroid rats [20, 23].

Increased xanthine oxidase (XO) expression and activation has been acknowledged in heart failure in both animals [74, 75] and humans [76]. Studies on the liver of hyperthyroid rats have proposed that XO is a key source of free radical production in hyperthyroidism [77]. Inhibition of XO has also been shown to decrease oxidative stress during thyrotoxicosis [78–80]. Besides, inhibition of XO was found to decrease TH-induced increase in serum NO_*x*_ as well as markers of lipid peroxidation, independent of the antioxidant enzymes. Additionally, this study suggested an association between XO inhibition and biosynthesis of THs [81]. To our knowledge, there is no data available about the direct role of XO in TH-induced oxidative stress in the heart. Recent data from our lab showed that the XO inhibitor, allopurinol, is not able to attenuate T4-induced cardiac hypertrophy, cardiac dysfunction, or hemodynamic changes [56], which may signify that XO is not involved in TH-induced cardiovascular changes.

There is growing evidence that cytochrome-P450 participates in the inception, progression, and prognosis of cardiovascular diseases including cardiac hypertrophy and heart failure in experimental animal models as well as in human patients [82, 83]. Analysis of differentially expressed genes in hyperthyroid-induced hypertrophied heart by cDNA microarray has revealed induction of cytochrome-P450 isoforms [10], implying a role of these oxidative enzymes in the development of oxidative stress in the heart following hyperthyroidism.

At low concentrations, catecholamines stimulate the heart by inducing Ca^2+^ movements, while at higher concentrations they can often result in cardiac dysfunction by provoking intracellular Ca^2+^ overload in cardiomyocytes. Additionally, numerous studies have reported that under stressful conditions excessive amounts of catecholamines become oxidized to form aminolutins and generate ROS. Oxidation products of catecholamines have been shown to cause coronary spasms, arrhythmias, and cardiac dysfunction, as previously reviewed [84]. In hyperthyroidism, increased adrenergic activity had been accredited to altered heart sensitivity, an increase in free catecholamines at the myocardial receptor site, or an increase in circulating catecholamines [85]. An association has been reported between T4-induced cardiac hypertrophy and the adrenergic nervous system [86]. Nevertheless, there are contradictory reports concerning the anticipatory nature of adrenergic inhibition in hyperthyroidism-induced cardiac hypertrophy [44, 55, 86–88]. As far as we know, no connection has been reported between the autoxidation of catecholamines and TH-induced oxidative stress in the heart.

Overall, potential sources for ROS generation in the hyperthyroid hearts could include mitochondria, NADPH-oxidase, NOS, and cytochrome-P450 as illustrated in Figure 1.

## 4. Cellular and Molecular Consequences of Increased Oxidative Stress in Hyperthyroid Hearts

In biological systems, oxidative damage of macromolecules such as lipids, proteins, and DNA has been proposed as a key indicator of oxidative stress [54].  Figure 2 demonstrates the cellular consequences of oxidative stress in hyperthyroid hearts. In hyperthyroidism, lipid peroxidation has been commonly used as an index of oxidative stress since polyunsaturated fatty acids are particularly vulnerable to ROS assault, and derivatives of lipid peroxidation can be simply assessed. As illustrated in Figure 2, the majority of studies show increased lipid peroxidation in the hyperthyroid heart. However, in some few instances there are inconsistencies among published results. For example, Gredilla et al. reported that endogenous levels of lipid peroxides were not altered by the hyperthyroid state although heart sensitivity to lipid peroxidation increased [14]. Also, hearts of older hyperthyroid rats showed increased lipid peroxidation; however, younger rats displayed no change [45]. These inconsistencies have been attributed to a range of factors, such as species, iodothyronine used, treatment duration, and/or the variability in the accuracies of the methods used for determination of lipid peroxidation. Regarding the latter, the method used for the evaluation of thiobarbituric acid reactive substances (TBARS) for instance is not always very accurate and may return results which can widely vary depending on the conditions used in the assay [54].

There are few data available regarding the impact of THs-induced oxidative stress on cardiac protein and DNA oxidation (Figure 2). Although it is obvious that hyperthyroidism induces protein oxidation in the heart, as indicated by increased protein-bound carbonyls content [18, 20], oxidative damage to genomic DNA, evaluated as 8-oxo-7,8-dihydro-2′-deoxyguanosine (8-oxodG), was inconsistent. 8-oxodG did not show any changes in the rat heart following 10-day T3 treatment [89]; however, a longer T4 treatment time (5 weeks) has been shown to decrease 8-oxo-dG levels in mouse hearts [14]. The lack of cardiac 8-oxodG increase has been explained by (1) interception of most of the H_2_O_2_ produced by different cellular sources by cytosolic antioxidants before it arrives at the nucleus, (2) lower susceptibility of nuclear DNA to ROS attacks due to extensive covering by proteins such as histones [90], and (3) rapid repair of 8-oxodG by a specific 8-oxoguanine DNA glycosylase/lyase [91], as well as enhancements in oxidative stress-induced increase in DNA oxidative damage repair [92]. In contrast to genomic DNA, mitochondrial DNA damage was found to be significantly higher in the hyperthyroid heart, and this has been mainly attributed to its localization near the principal ROS production site [14].

In summary, lipid peroxidation and oxidative protein damage could be considered the main cellular consequences of oxidative stress in hyperthyroid hearts. ROS-driven oxidation of membrane phospholipids and/or hydrosulfide-containing proteins can cause alterations in channel activity and changes in the membrane currents leading to electrophysiological alterations and contractile dysfunction observed in the hyperthyroid hearts [53]. Oxidative changes in lipids and proteins can also contribute to cellular damage, energetic deficit, and acceleration of cell death through apoptosis and necrosis [93]. Indeed, depressed cardiac contractility and enhanced apoptosis have been proposed to result in heart failure in hypertrophied myocardium following hyperthyroidism [94]. Recently, induction of apoptosis-related signaling has been coupled to increased oxidative stress in the hyperthyroid hearts [24].

ROS generation may result in a cellular redox imbalance, which is the key activator of some signaling pathways such as NF-E2-related factor-2 (Nrf-2) pathway [95]. This could modulate gene expression of a variety of redox-sensitive proteins such as Trx and peroxiredoxin (Prx), which are essential for cellular defense in opposition to oxidative stress as well as for cell survival [96–99]. In hyperthyroid rats that revealed cardiac hypertrophy and ventricular dysfunction after 4-week treatment of T4, Araujo et al. showed that oxidative stress in the myocardium induces adaptations in the GPx-GR and Trx-Prx systems through Nrf-2 activation [22] (Figure 3). Conversely, the same group showed that this pathway was not collaborating with the maintenance of redox balance after 2-week treatment of T4, when the same rats exhibited cardiac hypertrophy but preserved cardiac function [24]. In addition to its role in keeping redox homeostasis, Trx has also been involved in the repression of ROS-mediated pathological cardiac hypertrophy, signifying a cardioprotective action, as well as in the regulation of the cell survival pathway [100, 101]. THs are consistently known to induce cardiomyocyte hypertrophy [12–29]. ROS are vital to the initiation and continuation of numerous signal transduction pathways involved in growth and differentiation of cells [102]. In addition, ROS do not only regulate diverse transcription factors but also could be active as second messengers in coordinating several significant cellular functions, such as proliferation and apoptosis [103]. For instance, IGF-1 stimulates proliferation of cardiomyocytes through binding to its receptor, which is expressed in the heart at high levels [104]. Araujo et al. showed that in experimental hyperthyroidism expression of IGF-1 receptors can be regulated via changes in the cellular redox state, directing cardiomyocyte growth [19]. Additionally, IGF-1 could trigger the AKT1 (protein kinase B) signaling pathway, which is critically involved in cardiac growth regulation [105]. Notably, it has been reported that T4 promotes the AKT1 signaling pathway in the heart, which in turn contributes to the cardiac hypertrophy observed in this model [106]. Likewise, Araujo and coworkers found that both active-Akt and active-Akt/total-Akt ratio were significantly increased in the hearts of hyperthyroid rats with cardiac hypertrophy and ventricular dysfunction after 4-week treatment of T4 [20]. Interestingly, they strongly proposed H_2_O_2_ as a possible mediator for the activation of the AKT1 pathway, confirming a key role for oxidative stress in the activation of this signaling pathway in experimental hyperthyroidism [20]. This could be directly attained by H_2_O_2_ by changing conformation of protein and increasing vulnerability to phosphorylation or secondarily in inducing imbalance of redox status (GSH/GSSG ratio) [20]. Astoundingly, the same group showed decreased active- and total-Akt with no change in the active-Akt/total-Akt ratio in the same rats with cardiac hypertrophy but preserved cardiac function after 2-week treatment of T4 [24]. In this study, they indicated that decreased Akt expression was correlated with redox imbalance. However, the exact mechanisms responsible for the coordination of this effect remain to be defined [24]. Another important redox-sensitive pathway that is involved in cardiac growth and apoptosis is the mitogen activated protein kinase (MAPK) pathway including extracellular signal-regulated kinase (ERK1/2), Jun NH2-terminal kinase (JNK), and p38 MAPK. In effect, ERK1/2 activation was found to increase in response to increased oxidative stress in the hypertrophied hyperthyroid hearts with either preserved [16, 24] or deteriorated cardiac functions [22] without changes in JNK or p38 MAPK [16, 22]. Moreover, Araujo et al. [23] found that angiotensin-II receptor (AT1/AT2) gene expressions were enhanced in the hypertrophied hyperthyroid hearts. Importantly, they proposed that ROS/NO balance may be a key player in controlling the TH-induced cardiac hypertrophy mediated by the renin-angiotensin system. In a further study, the same group showed a positive impact of renin-angiotensin system blockade with an AT1-blocker, losartan, in the autonomic control of heart rate which was coupled with a decline in H_2_O_2_ levels, as well as with a decreased counterregulatory response of heme-oxygenase-1, and cardiac hypertrophy in experimental hyperthyroidism [66]. Yet there are contradictory reports concerning the inhibitory effect of AT1-blocker, losartan, in TH-induced cardiac hypertrophy. Kobori et al. [107, 108] reported a positive effect for losartan on T4-induced cardiac hypertrophy, while others reported negative effects [27, 86]. In agreement with these latter studies, unpublished data from our lab showed that losartan (5 mg/kg/day) administered by intraperitoneal injection before T4 for 2 weeks could not prevent the T4-induced cardiac hypertrophy in our model. Consistent with these results, Carneiro-Ramos et al. noticed that cardiac AT1 receptor expression did not change in the TH-induced cardiac hypertrophy. However, they found that cardiac expression of AT2 receptor is increased and that the AT2 receptor is a main player in the development of TH-induced cardiac hypertrophy [109]. In conclusion, redox-sensitive signaling such as IGF-1, AKT-1, and ERK1/2 was consistently found to increase in hyperthyroid hearts. Although these increases have been mainly associated with cardiomyocyte growth and cardiac hypertrophy (Figure 3), the possibility of being increased as a compensatory mechanism to protect the cardiomyocyte against oxidative stress and subsequent cell death cannot be excluded [110–112].

Hyperthyroid rats with cardiac hypertrophy and preserved cardiac function after 2-week treatment of T4 displayed increased Bax: Bcl-2 ratio, which signalizes a mitochondrial apoptotic pathway [24] (Figure 3). However, there were no changes in caspase-3 expression in the T4 rats. Since cardiac function is maintained at this time point, apoptosis is improbable. Furthermore, parameters assessed in that study were not sufficient to recognize the apoptotic mechanisms in the hyperthyroidism, but the collective results propose the activation of proteins implicated in decompensated cardiac remodeling which could progress to heart failure at later stages [24]. Consistent with these results, we previously have reported that increased ROS production in hyperthyroid hearts was not associated with increased caspases (caspase-8 and caspase-3) or apoptosis at stages of preserved cardiac function [16]. Mostly, this could happen at later stages of deteriorated cardiac function based on a recent report showing that depressed cardiac contractility and enhanced apoptosis have been proposed to result in heart failure in hypertrophied myocardium following hyperthyroidism [94].

## 5. Effects of Antioxidant Treatments on Thyroid Hormones-Induced Cardiac Hypertrophy and Associated Cardiac Dysfunction

Cardiac hypertrophy represents the most documented cardiomyopathy following hyperthyroidism in experimental animals. TH-induced cardiac hypertrophy has been described as relative ventricular hypertrophy that encompasses the whole heart (right ventricle (RV) and LV), and this was linked to contractile abnormalities in both ventricles [17]. The acceleration of oxidative stress, which takes place in experimental hyperthyroidism, proposes that ROS are key players in the cardiomyopathy frequently reported in this endocrine disorder [52]. The effectiveness of standard antioxidant treatments or other oxidative stress-protecting drugs on the THs-induced cardiac hypertrophy and/or associated cardiac dysfunction has been reported in several studies as shown in Table 2.

Among all antioxidants, vitamin E represents the most frequently used antioxidant in experimental hyperthyroidism. Vitamin E is a lipophilic and chain-breaking antioxidant that works by slotting into the lipid bilayer, where it can impede the development of lipid peroxides and carbonyl groups due to its ability to scavenge the alkyl, alcoxyl, and peroxyl radicals to finally inhibit lipid peroxidation as well as protein oxidation [20]. Asayama et al. reported that vitamin E protects against lipid peroxidation in hyperthyroid hearts independent of the changes in oxidative enzymes and antioxidant enzymes, without affecting the cardiac hypertrophy in this model. They also proposed that vitamin E would be helpful in preventing cardiac muscle damage in hyperthyroid subjects [25]. Similarly, Venditti et al. showed that vitamin E protects hyperthyroid heart against lipid peroxidation independent of the changes in antioxidant systems, without affecting the cardiac hypertrophy in this model. However, they indicated that vitamin E partially attenuated changes in* in vivo* heart rate as well as in* in vitro* action potential duration shortening of isolated RV papillary muscles. These functional changes have been proposed to be mediated, at least in part, through a membrane modification, probably related to increased lipid peroxidation [52]. In a further study, in addition to vitamin E, the same group also used N-acetylcysteine (NAC) and cholesterol. NAC is a classic antioxidant that can reduce the peroxidative processes due to its high capability of scavenging ^∙^OH radical and acting as a precursor and upregulator of GSH synthesis. On the other hand, cholesterol is not an antioxidant but is capable of inhibiting the peroxidative processes possibly through a mechanism that involves a decline in membrane fluidity [53]. Even though vitamin E, NAC, and cholesterol significantly decreased lipid peroxidation, only vitamin E and NAC were able to partially improve the TH-induced shortening in action potential duration. It was concluded that the antioxidant-sensitive shortening of action potential duration evoked by THs is probably independent of increased peroxidative processes in the sarcolemmal membrane [53]. The protective effect of vitamin E has been suggested to be due to its ability to protect the hydrosulfide-containing ion channel proteins, whereas the protective effect of NAC was attributed to its capability of increasing the competence of the vitamin E system upholding high concentrations of GSH [53]. In this latter study, in addition to improving the lipid peroxidation, vitamin E and NAC only increased total antioxidant capacity. None of the three drugs (vitamin E, NAC, and cholesterol) were able to attenuate the TH-induced cardiac hypertrophy [53]. At variance, in a series of studies, Araujo et al. showed that not only vitamin E improved the TH-induced cardiac dysfunction, but also it significantly decreased cardiac hypertrophy following hyperthyroidism [19, 20, 22, 23]. They revealed that vitamin E inhibits lipid peroxidation and protein oxidation and attenuates changes in oxidative and antioxidative enzymes and related redox-sensitive signaling such as IGF-1 receptors [19], AKT1 [20], ERK1/2 [22], NADPH-oxidase, NOS, and AT1 receptors [23]. On functional levels, vitamin E partially improved TH-induced changes in LV systolic pressure, but it did not affect LV diastolic pressure. Conversely, it normalized the positive (+*dP*/*dt*) and the negative (−*dP*/*dt*) pressure derivatives, which are more sensitive indicators of ventricular contractility and relaxation, respectively [19, 20]. Additionally, vitamin E significantly reduced organ (liver and lung) congestion, which is a hallmark of congestive heart failure [19, 20]. While vitamin E has been consistently reported to have positive effects on the TH-induced cardiac dysfunction, its effect on associated cardiac hypertrophy is not consistent. For reliability, effects of vitamin E on thyroid function should also be clearly reported in each individual study as vitamin E has been described to have an inhibitory action on the thyroid functions. Previous reports showed that vitamin E decreases T4 and T3 levels in euthyroid rats and propose that vitamin E reduces either the synthesis of T4/T3 or the conversion of T4 to T3 [2, 113].

Asayama et al. [44] investigated the effect of *β*-adrenergic blockers with different ancillary properties (carteolol: a *β*-blocker with partial agonist activity, atenolol: selective *β*_1_-blocker, and arotinolol: a *β*-blocker with weak *α*-blocking activity) on lipid peroxidation in the cardiac muscle of hyperthyroid rats. Although atenolol alone was able to inhibit the T4-induced acceleration of lipid peroxidation and mitochondrial hypermetabolism in the hearts of these rats, it did not affect the increased cardiac mass in this model [44]. Likewise, neither nonselective inhibitor of all NOS isoforms [27] nor selective inhibitors of iNOS [28] or nNOS [29] were able to attenuate the T4-induced cardiac hypertrophy in rats. Furthermore, tempol (4-hydroxy-2,2,6,6-tetramethyl piperidinoxyl), a stable metal-independent and cell membrane-permeable low-molecular-weight SOD mimetic drug, did not improve cardiac hypertrophy in hyperthyroid rats [26]. Moreover, carvedilol, which is a nonselective vasodilating *β*-blocker working on *β*_1_-, *β*_2_-, and *α*_1_-adrenoceptors with a potent antioxidant action possibly due to its ability to (1) scavenge O_2_^∙−^, (2) inhibit O_2_^∙−^ production, (3) attenuate lipid peroxidation, and (4) spare the consumption of endogenous antioxidants [16], could not decrease the cardiac hypertrophy in hyperthyroid rats [55]. Consistent with these reports, we have recently shown the inability of several antioxidants, including allopurinol (xanthine oxidase inhibitor), apocynin (NADPH-oxidase inhibitor), L-NIO (nitric oxide synthase inhibitor), or Mito-TEMPO (mitochondria-targeted antioxidant), to recover the T4-induced cardiac hypertrophy in mice [56]. Nevertheless, this does not completely rule out the contribution of ROS in the development of T4-induced cardiac hypertrophy. Our previous findings demonstrate that pravastatin, by inhibiting myocardial Rac1 (a major component of NADPH-oxidase), did not decrease the gross heart weight but significantly decreased the cardiomyocyte size to a level that was still higher than control, thus indicating a partial role for ROS in this response [17]. Changes in cardiomyocyte size are generally followed by consistent changes in the heart weight; however, this may not happen in some cases. Both increased [15, 114] and decreased [17, 86] cardiomyocyte sizes without corresponding changes in the gross heart weight have been reported. Our recent results also showed that treatment with L-NIO exhibited a strong trend towards improving the LV functions as evident by increased LV ejection fraction and fractional shortening; however, these increases did not reach significance [56]. On the contrary, inhibition of NADPH-oxidase by apocynin significantly improved T4-induced LV systolic dysfunction in mice. Interestingly, this happened in the absence of any effects for apocynin on the cardiac mass, which means that NADPH-oxidase plays a major role in the T4-induced LV dysfunction regardless of the cardiac hypertrophy. This also indicates for the first time that T4-induced LV dysfunction is independent of the development of cardiac hypertrophy [56]. In contrast to apocynin, in our previous study [17] pravastatin significantly decreased myocardial Rac-GTPase activity; however, it did not show any improvement in the LV systolic function. The reasons for this discrepancy are not clear. Still, there are apparent differences in the nature and the dose of both drugs which were used in the two studies.

As mentioned above, the T4-induced RV hypertrophy is linked to marked contractile abnormalities, including decreased contraction/relaxation times, a negative force-frequency relationship, and a blunted *β*-adrenergic response [17, 56]. In our hands, none of the antioxidant treatments (pravastatin, allopurinol, apocynin, L-NIO, and Mito-TEMPO) were able to reverse these T4-induced effects* ex vivo*. Although apocynin had a trend to show better responses in relation to other drugs, these responses were insignificant compared to those of the T4 muscles [56]. The LV and RV have differences in structure, function, and response to stress and disease [115]; hence, their differential responses to treatment could be expected. In this regard, improved LV but not RV function has been reported in human patients following treatment with carvedilol [116]. Another possible explanation for the different responses of antioxidants on the LV and RV is that LV function was assessed* in vivo*, while RV contractile parameters were evaluated* ex vivo*. Thus, the effect of antioxidant treatments on the* in vivo* RV function remains to be elucidated.

## 6. Conclusion and Future Perspectives

Elevated oxidative stress is a principal outcome in the hearts of experimental animals following hyperthyroidism. Our data along with data from several investigators show that oxidative stress is either not or only partially involved in the TH-induced cardiomyocyte hypertrophy. In contrast, oxidative stress seems to be a key player in the TH-induced LV dysfunction. Recently, our group was able to disclose one of the secrets of this process and show for the first time that NOS and more significantly NADPH-oxidase are major determinants in this process regardless of cardiac hypertrophy [56], as shown in Figure 4. In general, oxidative and nitrosative stresses can result in cardiac dysfunction through (1) desensitization of contractile protein, (2) changes in cellular energetics, (3) alterations in excitation-contraction coupling, (4) variations in myofilament calcium responsiveness, and/or (5) endothelial dysfunction [67, 117]. However, the precise cellular, biochemical, and molecular mechanism(s) behind the improving effects of antioxidants on cardiac dysfunction following hyperthyroidism remain to be examined. In spite of elevated oxidative stress in the heart being linked to increased THs levels several decades ago, a clearly defined association between this increased oxidative stress and cardiac dysfunction in experimental hyperthyroidism still represents an undisclosed story.

Thyroid disease is rather prevalent. Recent estimations imply that it affects about 9–15% of the adult female population and a lesser proportion of adult males. Nonetheless, with advancing age, particularly beyond the eighties, the occurrence of disease in males increases to be equivalent to that of females [11]. Heart failure occurs in 6–15% of hyperthyroid patients [118]. Timely and efficient treatment of cardiac manifestations in hyperthyroid patients is essential because cardiovascular complications comprise most of the deaths in these patients. Managing heart failure in hyperthyroid patients is complicated because symptoms of heart failure may be coupled with assorted entities [118]. It has been reported that the improvement of thyroid dysfunction must be the initial procedure applied in the hyperthyroid patients with heart failure. Ultimate treatment of hyperthyroidism is frequently achieved to improve cardiac function [118]; however, increased cardiac mortality has been reported to be a trend in the treated hyperthyroid patients [119]. Therefore, the exact way to treat hyperthyroid patients with heart failure remains incompletely understood. Forcing the application of new therapies such as NOS or NADPH-oxidase inhibitors along with antithyroid drugs or other potentially effective drugs in the treatment of THs-induced cardiac hypertrophy would carry a large promise for the hyperthyroid patients. Examining the effectiveness of these combination therapies in attenuating TH-induced cardiomyopathy as well as recognizing their cellular and molecular mechanisms in experimental models of hyperthyroidism needs a lot of effort in the years to come. Validating the results of these preclinical studies in both small and large scale clinical trials should also be considered in order to give the hope of life for millions of people who are suffering from the hyperthyroidism and related heart problems.

## Figures and Tables

**Table 1:** Changes of endogenous antioxidants in the hyperthyroid hearts.

Antioxidant	Increased	No change	Decreased
Mn-SOD	[25, 43–45]	[46]	
Cu, Zn-SOD	[18, 19]	[43, 45, 46]	[25, 44, 47]
Total SOD	[19, 45, 46]		[26]
CAT		[18, 19, 25, 45, 46]	[26, 43, 44]
GPX	[22, 47]	[45, 48]	[26, 43–46, 53]
GR	[22]	[48, 53]	[26]
GST	[18, 19]		
GSH	[49]	[14, 24]	[18–20, 22, 50, 53]
Trx and Trx reductase	[22]	[24]	
Prx	[22]		
Vitamin C			[20]
Vitamin E	[45, 46, 48]	[53]	
Co-Q9		[51]	[46]
Co-Q10		[46]	
*C*_A_		[52, 53]	[20, 48]

Mn: manganese; SOD: superoxide dismutase; Cu: copper; Zn: zinc; CAT: catalase; GPx: glutathione peroxidase; GR: glutathione reductase; GST: glutathione-S-transferase; GSH: glutathione; Trx: thioredoxin; Prx: peroxiredoxin; Co-Q: coenzyme-Q; *C*_A_: total antioxidant capacity.

**Table 2:** Effects of antioxidants or drugs protecting against oxidative stress on thyroid hormone-induced cardiac hypertrophy and associated cardiac dysfunction.

Drug	Mechanism	Cardiac hypertrophy	Cardiac dysfunction	Reference
Vitamin E	Inhibits lipid peroxidation independent of changes in oxidative or antioxidant enzymes	No change	NA	[25]
	Inhibits lipid peroxidation and increased total antioxidant capacity	No change	Partially improved shortened APD of isolated RVPM *in vitro *	[52, 53]
	Inhibits lipid and protein oxidation and attenuates changes in oxidative, antioxidative enzymes and related signaling for example IGF-I, AKT, ERK 1/2, NADPH-oxidase, NOS, and AT1R	Decrease	Normalization of ventricular (+/−) *dP*/*dt* and inhibition of organ (liver and lung) congestion, which is a marker of heart failure	[19, 20, 22, 23]

Atenolol	*β*-blocker suppresses mitochondrial hypermetabolism and oxidative stress	No change	NA	[44]

NAC	Antioxidant inhibits lipid peroxidation and increases total antioxidant capacity	No change	Partially improved shortened APD of isolated RVPM *in vitro *	[53]
Cholesterol	Inhibits lipid peroxidation	No change	No change	
L-NAME	Nonspecific inhibitor of all NOS isoforms (eNOS, iNOS, and nNOS)	No change	NA	[27]

AG	Specific inhibitor of iNOS	No change	NA	[28]

7-NI	Specific inhibitor of nNOS	No change	NA	[29]

Tempol	Cell membrane-permeable low-molecular-weight SOD mimetic drug	No change	NA	[26]

Carvedilol	Mixed *α*, *β*-blocker with antioxidant activities	No change	NA	[55]

Pravastatin	Inhibits active Rac1, a major component of NADPH-oxidase complex	No change^*∗*^	No change	[17]

Allopurinol	Xanthine-oxidase inhibitor	No change	No change	[56]
Apocynin	NADPH-oxidase inhibitor	No change	Significant increase in LV EF and FS	
L-NIO	Nonspecific inhibitor of all NOS isoforms (eNOS, iNOS, and nNOS)	No change	Strong trend to increase LV EF and FS but did not reach significance	
Mito-TEMPO	Mitochondria-targeted antioxidant	No change	No change	

APD: action potential duration; RVPM: right ventricular papillary muscle; IGF-1: insulin-like growth factor-1; ERK: extracellular regulated kinase; NADPH: nicotinamide adenine dinucleotide phosphate; NOS: nitric oxide synthase; AT1R: angiotensin receptor type-1; +*dP*/*dt*: positive pressure derivative; −*dP*/*dt*: negative pressure derivative; NAC: N-acetylcysteine; eNOS: endothelial nitric oxide synthase; iNOS: inducible nitric oxide synthase; nNOS: neuronal nitric oxide synthase; L-NAME: N^w^-nitro-L-arginine methyl ester; AG: aminoguanidine; 7-NI: 7-nitroindazole; SOD: superoxide dismutase; L-NIO: N^5^-(1-iminoethyl)-L-ornithine dihydrochloride; Mito-TEMPO: (2-(2,2,6,6-tetramethylpiperidin-1-oxyl-4-ylamino)-2-oxoethyl) triphenylphosphonium chloride; LV: left ventricular; EF: ejection fraction; FS: fractional shortening; ^*∗*^no change in gross heart weight or heart weight/body weight, but there was a partial but significant decrease in cardiomyocyte size; NA: not assessed.
